# Immunomodulatory role of oral microbiota in inflammatory diseases and allergic conditions

**DOI:** 10.3389/falgy.2023.1067483

**Published:** 2023-02-17

**Authors:** Carlos M. Moreno, Ellie Boeree, Claudia M. Tellez Freitas, K. Scott Weber

**Affiliations:** ^1^ Department of Microbiology and Molecular Biology, Brigham Young University, Provo, UT, United States; ^2^ College of Dental Medicine, Roseman University of Health Sciences, South Jordan, UT, United States

**Keywords:** oral microbiome, allergy, local inflammation, systemic inflammation, microbiota, oral microbiota

## Abstract

In recent years, the interplay between oral microbiota and systemic disease has gained attention as poor oral health is associated with several pathologies. The oral microbiota plays a role in the maintenance of overall health, and its dysbiosis influences chronic inflammation and the pathogenesis of gum diseases. Periodontitis has also been associated with other diseases and health complications such as cancer, neurogenerative and autoimmune disorders, chronic kidney disease, cardiovascular diseases, rheumatic arthritis, respiratory health, and adverse pregnancy outcomes. The host microbiota can influence immune cell development and immune responses, and recent evidence suggests that changes in oral microbiota composition may also contribute to sensitization and the development of allergic reactions, including asthma and peanut allergies. Conversely, there is also evidence that allergic reactions within the gut may contribute to alterations in oral microbiota composition. Here we review the current evidence of the role of the oral microbiota in inflammatory diseases and health complications, as well as its future relevance in improving health and ameliorating allergic disease.

## Introduction

**Figure 1:**
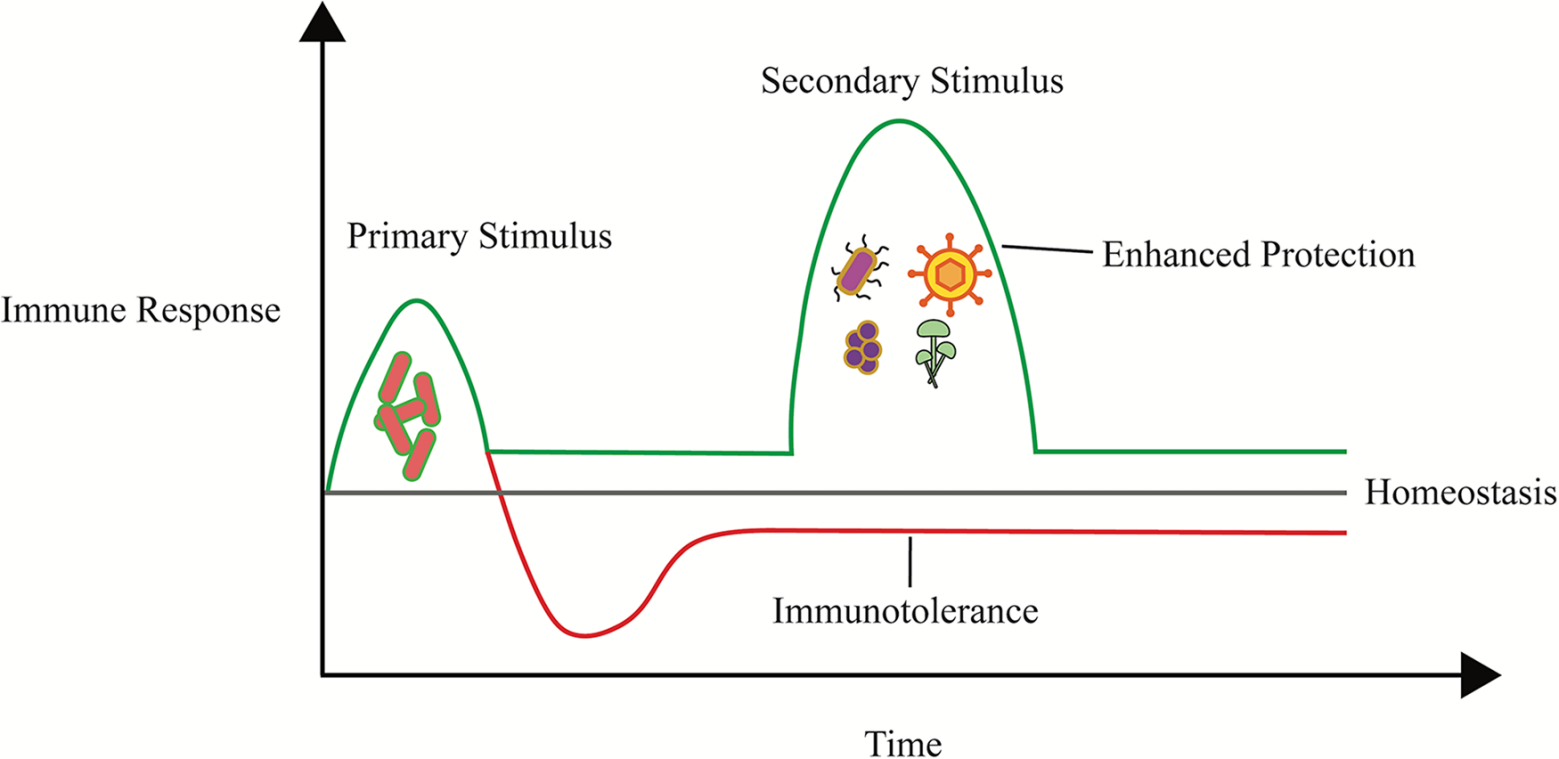
Host microbiota prime immune cells against pathogens or promote immunotolerance. Graph depicting immune training in which a primary stimulus either primes immune responses to protect against or tolerate microbial challenge.

The human microbiota is comprised of all the microbes (prokaryotes, archaea, and viruses) within the body, and it plays a homeostatic role in the development of a healthy immune system. Microbiota interact with host innate and adaptive immune cells in a way that facilitates immune training and tolerance towards pathogenic and commensal bacteria (Figure 1) (1). Additionally, interactions between microbiota and the host immune system are necessary for the proper development of immune cell populations and lymphoid organ development as demonstrated by early studies using germ-free (GF) mice (1–4). For example, population levels of adaptive immune cells including αβ and γδ intraepithelial lymphocytes (IEL), T regulatory (Treg) cells, and T helper 17 cells (Th17) are significantly reduced in the intestines of GF mice (2, 3, 5). The absence of microbiota within the intestines of GF mice also leads to reduced IgA antibody levels (6). Impairment of lymphoid tissue structure development and pathologies within the thymus of GF mice have also been observed (4, 7). Immune cell populations and IgA levels within the gut can be rescued by *de novo* microbial colonization (2, 6). The entire gastrointestinal (GI) tract is in constant contact with various microbes, most of which enter through the mouth (8). The oral cavity is home to a diverse community of microorganisms including bacteria, fungi, protozoa, and viruses (9, 10). Approximately 700 bacterial species have been identified within the oral cavity of humans using molecular analysis, and individuals typically contain approximately 100–200 species (9–11).

Throughout the GI tract, microbes are typically compartmentalized to the mucosal layer except for the oral cavity in which bacteria may form biofilms on the surfaces of teeth (12). Microbes may break the mucosal barrier during infection when a pathogenic strain invades underlying soft tissue, such as bacteria penetrating the periodontal epithelium surrounding teeth (13). Within the oral cavity, antimicrobial peptides, IgA, and mucin serve as a chemical barrier against infection, much like the mucosal immune system within the intestines (8, 13). However, epithelia in the mouth are multilayered, vary in their permeability and function, and include keratinized and non-keratinized epithelia which differs from the characteristics of intestinal epithelia (8, 13). The multiple layers of oral epithelia provide added protection against invasive pathogens and may contribute to immune tolerance *via* limiting antigen recognition (8). The mucosal immune system also includes mucosa associated lymphoid tissue (includes the salivary glands and tonsils, the lymphoid follicles, and draining lymph nodes) where most antigen stimulation and clonal expansion of lymphocytes occurs (13, 14). Keratinocytes within the oral cavity recognize bacteria, fungi, protozoa, and viruses through a variety of pattern recognition receptors, including toll-like receptor 2 (TLR2), TLR4, nucleotide-binding oligomerization domain 1 (NOD1), and NOD2 (8, 15). Upon recognition of pathogens, keratinocytes release inflammatory cytokines and chemokines that attract immune cells, such as T cells and natural killer (NK) cells, to the site of infection and create an inflammatory environment (15). Being immunocompromised and the use of antibiotics and antifungals are factors that can lead to infections by opportunistic bacterial and fungal pathogens within the oral cavity (16). Additionally, poor oral hygiene can lead to alterations in microbiota composition that disrupt their metabolic and functional roles (dysbiosis) and subsequent infection.

The interactions between the host immune system and the microbiota within the oral cavity have been the focus of study in recent publications (17–19). Gum disease, particularly periodontitis, is associated with several pathologies and health complications, such as adverse pregnancy outcomes, cardiovascular disease, pulmonary disease, inflammatory bowel disease (IBD), cancer, and type 2 diabetes mellitus (20–23). A recent study linked *Porphyromonas gingivalis*, a gram-negative bacterium that is one of the keystone pathogens of periodontitis, with Alzheimer's disease in humans, and other researchers are investigating this association in mouse and rat models (24–28). In another study, ectopic colonization of oral microbiota, particularly *Klebsiella* species, was found to promote Th1 polarization and inflammation within the gut (29). Chronic inflammation due to infection, such as chronic periodontitis, or trauma can lead to several clinical consequences as inflammatory mediators drive further tissue damage and increase the risk of hypertension, hyperglycemia, type 2 diabetes, chronic kidney disease, depression, neurodegenerative diseases, and various types of cancer (30). These findings suggest that oral microbiota may influence inflammatory responses locally and systemically. The potential role of oral microbiota influencing other inflammatory conditions such as allergies is a novel field of research with an impact on health and disease (31). Most recently, evidence suggests that changes in oral microbiota composition are also associated with sensitization and the development of allergic reactions, including asthma and peanut allergies (31–34). Of note, there is evidence that allergic reactions within the gut may contribute to oral dysbiosis in mice (35). Herein, we review the current evidence of the role of the oral microbiota in inflammatory diseases and allergic conditions, as well as its future relevance in ameliorating these inflammatory diseases and allergic conditions.

## Host microbiota influence immune system development

The host microbiota is comprised of the various microorganisms found and distributed in distinct parts of the body. Commensal microbiota are recognized by the innate immune system through the interactions of microbe-associated molecular patterns (MAMPs) and pattern recognition receptors (PRRs) expressed in most immune cells, contributing to the training of these immune cells to recognize and eliminate pathogens or promote immunotolerance (Figure 1) (36, 37). Upon recognition, immune cells and other cell types which express PRRs, including epithelial cells, may release cytokines that influence immune responses. Some common innate immune cell types that play roles in inflammatory and allergic diseases include mast cells, basophils, innate lymphoid cells (ILC), macrophages, and dendritic cells. Heterologous innate immune training is a phenomenon in which innate immune cells encounter a primary stimulus that enables them to better eliminate pathogens of different genera or even domains of life (37). For example, the administration of the Bacillus Calmette–Guerin vaccine, a vaccine against tuberculosis, to humans and mice has been found to provide protection against viral, bacterial, fungal, and parasitic infections *via* trained immunity (37, 38). Dendritic cells (DCs) are phagocytic innate immune cells that can exhibit inflammatory and anti-inflammatory profiles. They play roles in mucosal immunity as they sample antigens and activate lymphocytes after antigen recognition, processing, and peptide presentation on MHC-class II molecules to the lymphocytes. Recognition of certain MAMPs can influence the activation profiles and function of DCs (39). For instance, recognition of the β-glucan wall of *Candida albicans* promotes a tolerogenic state for those DCs while recognition of *Mycobacterium tuberculosis* promotes an inflammatory profile (37, 39). Exposure of DCs to bacterial antigens has also been reported to protect against amoeba infection (40). Another example of immune training is macrophages stimulated *via* TLR signaling are primed towards anti-inflammatory profiles producing lower levels of inflammatory cytokines, such as IL-6 and reactive oxygen species, and exposure to β-glucans promotes cell viability (41–44).

The adaptive immune response, comprised of T cells and B cells, complements innate immunity and is essential in the fight against infection. T cells drive cell-mediated and humoral-mediated immune responses, and their responses are influenced by their interactions with commensal microbiota. For example, microbiota-induced IL-1β production in the intestinal lamina propria induces steady-state differentiation of T helper type 17 (Th17) cells (45, 46). For both the innate and adaptive immune responses, commensal microbiota provide signals that keep the host's immune responses alert for invoking effective immunity when needed. These microorganisms also produce different metabolites and have specific genetic signatures that when interacting with the host have a homeostatic role in influencing the host's organ development, metabolism, and immune response (47–49). Studies in the gut looking at the interaction between microbiota and its host's immune cells suggest that such communication is more prominent at mucosal surfaces (50). One model supporting this communication and the role of the microbiota interactions with the host mucosal immune system is the GF mouse model. These mice have altered hepatic metabolic pathways, poor development of their gut-associated lymphoid tissue (GALT), smaller Peyer's patches, mesenteric lymph nodes, and impaired thymus development (51–54). Together these reported observations suggest that GF mice present underdeveloped immune systems and responses due to the absence of commensal microbiota. Additionally, GF mice colonized with mouse microbiota at birth no longer have altered secondary immune organs (55, 56). Bacterial metabolites such as short chain fatty acids (SCFAs), tryptophan, and retinoic acid (RA) can be detected by the host and influence hematopoiesis, immune responses, and allergic reactions (57–59). Bacterial antigens such as polysaccharide A produced by some commensal bacteria such as *Bacteroides fragilis* can also have an immunotolerant effect in the host by inducing IL-10 production and expanding Treg cells (60).

## The oral microbiota affects inflammatory diseases

**Figure 2:**
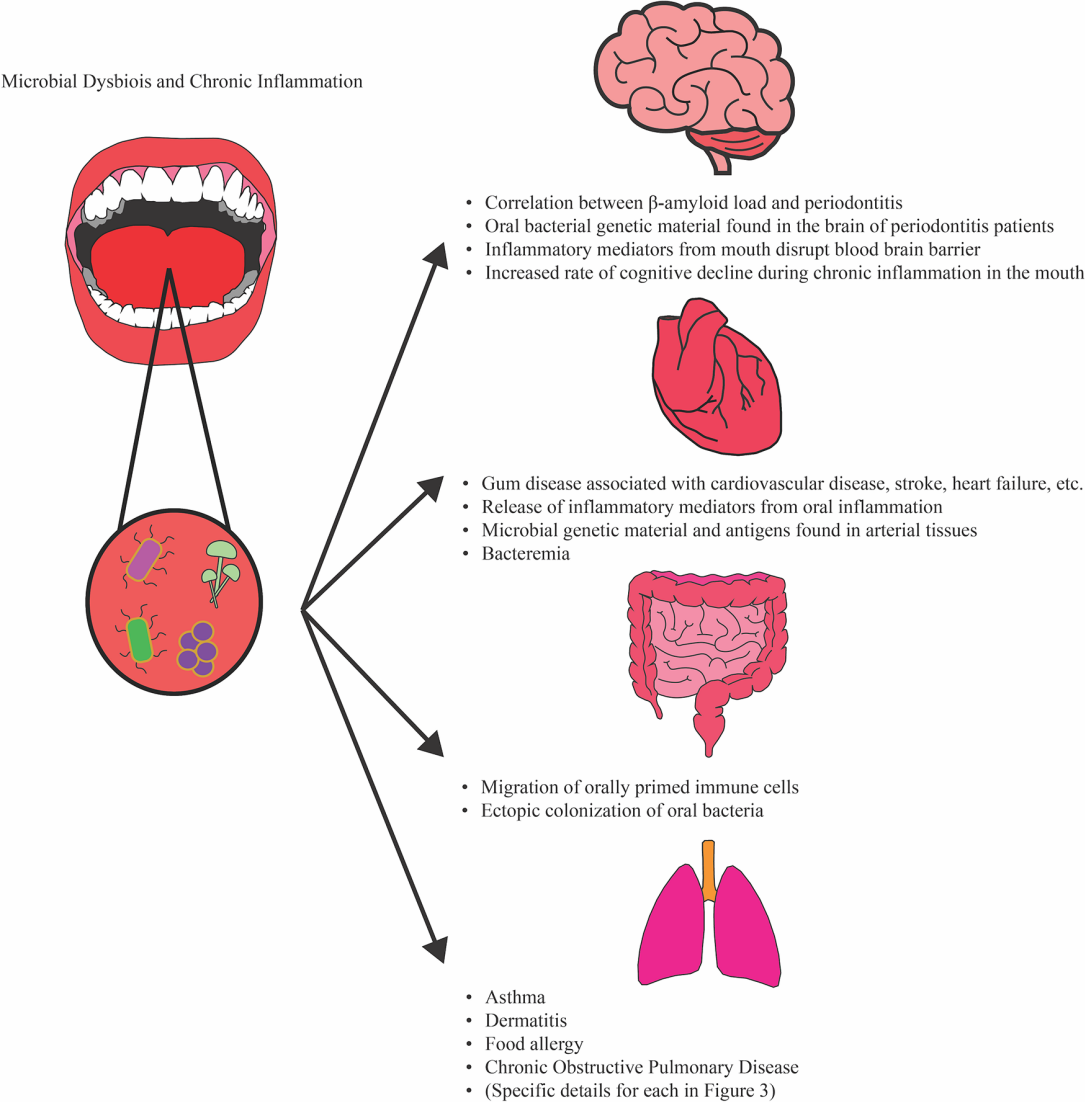
Associations between oral dysbiosis/chronic inflammation and pathologies in distant organs. Oral dysbiosis and chronic inflammation is associated with inflammatory conditions in distant organs, such as the brain, heart, intestines, and lungs. Associations between oral microbiota composition and allergy, dermatitis, asthma, and COPD development have also been reported and are covered in more detail in Figure 3. In the brain, the presence of inflammatory mediators and bacterial antigens may increase the risk of Alzheimer's disease progression and severity. Ectopic colonization and migration of orally primed immune cells have been found to exacerbate colitis in mice. Microbial genetic material and antigens have been found in arterial tissue, and chronic periodontitis have been associated with increased risk of cardiovascular diseases.

Recent evidence suggests that microbial dysbiosis and chronic inflammation within the oral cavity influences systemic inflammation and inflammatory diseases (Figure 2) (20). Of note, increased colonization of oral microbiota within the intestines has been reported in patients suffering from colorectal cancer, HIV, and treatment-naïve Crohn's disease (11, 29, 61–64). Several meta-analyses also indicate that patients with inflammatory bowel disease (IBD) are at higher risk of developing periodontitis and dental caries (65–67). These recent findings suggest that oral microbiota may impact diseases located in other organs and vice versa. In a study that investigated the interplay between oral microbiota and inflammation in the gut, Atarshi et al. reported that increased levels of *Rothia*, *Streptococcus*, *Neisseria*, *Pervotella*, and *Gemella* bacteria, typical oral microbiota, were found in the fecal microbiota of patients with inflammatory intestinal and liver diseases such as ulcerative colitis, primary sclerosing cholangitis, gastroesophageal reflux disease, and alcoholism (29). They also observed that colonization of *Klebsiella*, originally isolated from the saliva of a patient with Crohn's disease, within the intestines of mice induced inflammatory Th1 responses demonstrating that oral microbiota are potential promoters of inflammation (29). A study by Kitamoto et al. investigated how periodontitis and oral dysbiosis influences inflammation within the gut using ligature-induced periodontitis and dextran sodium sulfate (DSS)-induced experimental colitis mouse models (68). Their work demonstrated how ligature-induced periodontitis alone was able to induce the infiltration of Th17 cells, B cells, and γδ Τ cells in the lamina propria of the intestines. The increase in Th17 cells, accompanied by an increase in Th1 cells, was enhanced in oral ligature and DSS treated mice as validate with flow cytometry. Their study shows that oral pathobiont-specific effector memory T cells are able to migrate from cervical lymph nodes to the intestines and exacerbate colitis in mice (Figure 2) (68). These observations illustrate how the microbiota and immune responses in both the mouth and gut are intertwined and influence each other.

Oral dysbiosis and inflammation also has implications in neurodegenerative conditions, such as Alzheimer's disease. Recent studies have shown that chronic periodontitis is positively correlated with Alzheimer's disease, and investigators have determined that chronic periodontitis induces the secretion of inflammatory mediators, including C-reactive protein and IL-6 which may disrupt the blood-brain barrier and contribute to neuroinflammation (69, 70). Additionally, amyloid-β load in the brain of Alzheimer's patients correlates positively with periodontitis (71). A recent study analyzing publicly available data from the National Health and Nutrition Examination Survey performed by the CDC's National Center for Health Statistics found that Alzheimer's diagnoses and associated deaths correlated positively with levels of antibodies against *P. gingivalis* (72). Further research is needed to determine if *P. gingivalis* plays a causal role in the development and progression of Alzheimer's disease. In 2016, Ide et al. investigated whether chronic periodontitis was associated with both increased dementia severity and cognitive decline in Alzheimer's disease patients during a 6-month longitudinal study (24). They assessed the cognition and measured serum IL-10 and inflammatory markers (C-reactive protein, TNFα) of 60 patients with mild and severe Alzheimer's disease at the beginning of the study and at 6 months. They found that periodontitis was associated with a 6-fold increase in the rate of cognitive decline at the end of the 6-month period independent of baseline cognitive state. They also report a fall in serum IL-10 levels (*p* = 0.047) and a modest but not statistically significant increase in C-reactive protein was associated with the presence of periodontitis at baseline (24). These observations from these groups brings to question whether therapeutics to treat periodontitis reduces the risk of heightened disease progression in Alzheimer's disease.

Associations between oral dysbiosis and cardiovascular disease is another topic currently being investigated. There are certainly positive epidemiological associations between periodontitis and higher prevalence of subclinical cardiovascular disease, coronary artery disease, stroke, heart failure, and peripheral artery disease, many of these conditions probably caused by mechanical disruption of oral biofilms which lead to bacteremia (73). This hypothesis is supported by the fact that the DNA and RNA of periodontal pathogens have been found in atherothrombotic tissue (73–76). One possible mechanism for the ectopic colonization of oral bacteria in arterial tissue involves DCs which play a role in the initiation of the inflammatory response in periodontitis and direct the functions of T cells (77). Studies have observed the colocalization of a myeloid DC marker, CD209, and *P. gingivalis* minor fimbria protein, mfa-1, in the atherosclerotic plaques of deceased coronary artery-disease patients (77). *P. gingivalis* mfa-1 targets CD209 for entry into DCs and promotes survival within these cells, and the number of CD209+ DCs circulating in the periphery increase during periodontitis (77–80). Thus, inflammation and pathogen burden within the mouth may lead to further complications in cardiovascular disease. As mentioned above, inflammatory mediators, such as C-reactive protein and IL-6, are also elevated in the serum of periodontitis patients (81). C-reactive protein levels are especially higher in patients with periodontitis and cardiovascular disease than healthy patients or patients with either condition (73).

As demonstrated in the studies above, the oral cavity, along with its microbiota and inflammatory responses, is not an isolated region. Immune responses that initiate locally in the gingiva or mucosal epithelia of the mouth and develop into chronic inflammation may influence inflammatory responses in other regions of the body. Thus, further investigation on how treatment of oral inflammation impacts other inflammatory diseases can provide novel approaches to treating conditions such as Crohn's disease, cardiovascular disease, and Alzheimer's disease (Figure 2). Recently, our group performed a meta-analysis on publicly available RNAseq data to identify inflammatory mediators of periodontitis that contribute to local and systemic inflammation and potential drugs that can be repurposed to reduce inflammation (82). By performing analysis of differentially expressed genes and pathways, we determined that FDA-approved drugs that target IL-6 receptor, RANKL, and IFNα/β receptor were among our top candidates to reduce chronic inflammation during periodontitis. However, further investigation in the efficacy of these drugs in reducing inflammation during chronic periodontitis and ameliorating disease progression is needed.

## Oral microbiota composition may influence allergies and respiratory disease development

Allergy and hypersensitivity are the result of over responsiveness of the immune system to an otherwise harmless antigen, and chronic allergic inflammation can lead to serious health complications including altered organ function and increased risk of infection in affected sites (83). Allergic diseases, such as respiratory and food allergies, have heterogeneous inflammatory pathologies, characterized by specific dysregulated immunological responses (84). Allergic diseases can be categorized into three temporal phases: early-phase reactions which are induced within seconds of antigen exposure, late-phase reactions which occur within a few hours, and chronic allergic inflammation which persists due to persistent antigen exposure (83). It is during chronic allergic inflammation when tissue restructuring may occur. For example, nasal polyps may appear in patients with allergic rhinitis, and impaired barrier function in the upper airways of allergic rhinitis patients may render them susceptible to chronic sinus infections (83, 85, 86). In patients with asthma, increased number of goblet cells, increased deposition of extracellular matrix molecules in the lamina reticularis, increased number and function of smooth muscle cells, and increased vascularity are some of the changes that can occur in the respiratory tract (83, 87–89). Additionally, levels of tissue-resident immune cell populations, such as ILCs, mast cells, and T cells, typically increase (83, 90). As described above, oral dysbiosis has many implications in the development of chronic inflammatory conditions that influence the mouth and possibly other organs. Thus, it is reasonable that alterations in the oral microbiota composition and, consequently, oral mucosal immunity may influence allergy development locally or in distinct parts of the body. However, research investigating associations between oral inflammation and dysbiosis is still a novel field, and much more work is needed to determine causal relationships. In this section, we highlight some of the most recent findings on the relationship between oral microbiota composition and allergic respiratory disease development. A summary of the studies mentioned in this section can be found in Table 1 which lists information such as the disease studied, the ages of subjects, and oral microbiota implicated in allergic disease development.

**Table 1:** Summary of recent studies that identify oral microbiota associated with allergic disease development.

Disease(s)	Age of subjects	Number of subjects	Implicated microbiota*	Method	References
Allergy/Asthma	Infants and children (3 months to 7 years)	Allergy, *n* = 47 Control, *n* = 33	Bacteroides ↑ Prevotella ↑ Alloprevotella ↑ Gemella haemolysans ↑ Gemella sanguinis ↑ Streptococcus sp. ↑ Staphylococcus ↑ Lactobacillus gasseri ↓ Lactobacillus crispatus ↓	16S rRNA gene sequencing	Dzidic et al, 2018
Atopic dermatitis (AD)	Toddlers (1–3 years) Children (4–12 years) Teenagers-Adults (13–59)	AD oral, *n* = 84; skin, *n* = 121 Control oral, *n* = 77; skin *n* = 105	Klebsiella ↑ Halomonas ↑ Prevotella ↓ Acinetobacter ↑	16S rRNA gene sequencing	Li et al, 2019
Asthma	Children and Adults (8–21)	Asthma, *n* = 57 Control, *n* = 57	Veillonella ↑ Streptococcus ↓	16S rRNA gene sequencing	Espuela-Ortiz et al, 2019
Asthma	Adults	Atopic asthma, *n* = 32 Atopic nonasthmatic, *n* = 18 Nonatopic control, *n* = 16	Streptococcus ↓ Moraxella ↑ Neisseria ↑ Aggregatibacter ↓	16S rRNA gene sequencing	Durack et al, 2020
Food allergy	Median age = 10 years Interquartile range = 7–14	Allergy, *n* = 56 Control, *n* = 49	Prevotella ↓ Veillonella ↓ Neisseria ↑	16S rRNA gene sequencing	Ho et al, 2021

* Not all oral microbiota implicated in the disease of interest are listed due to space.

A key finding from the human microbiome project was that there is not a standard healthy microbiota taxonomic composition, but that “healthy” microbiota compositions were best predicted by the molecular functions of the strains found in the population (91, 92). The most common oral bacterial taxa found in healthy individuals are Firmicutes, Proteobacteria, Actinobacteria, Fusobacterium, and Bacteriodetes (9, 93). Recent studies have investigated alterations in oral-pharyngeal microbiota composition and its associations with allergy development, as well as asthma development. An important distinction between allergy and asthma is worth noting here. Asthma is a chronic respiratory disorder that manifests as episodes of wheezing, coughing, and shortness of breath, and severe episodes can sometimes lead to irreversible decline in lung function (94). Atopy, or the genetic tendency to develop allergic diseases such as rhinitis, and specific allergies have been associated with increased risk of developing asthma. However, not all asthmatic patients exhibit elevated allergic responses (94). Thus, allergy and asthma development are distinct though they are both inflammatory diseases and share some common risk factors. Some of the common risk factors between allergic sensitization and asthma development include race, sex, heredity, pollution, passive smoking, obesity, and respiratory viral infection (94, 95).

**Figure 3:**
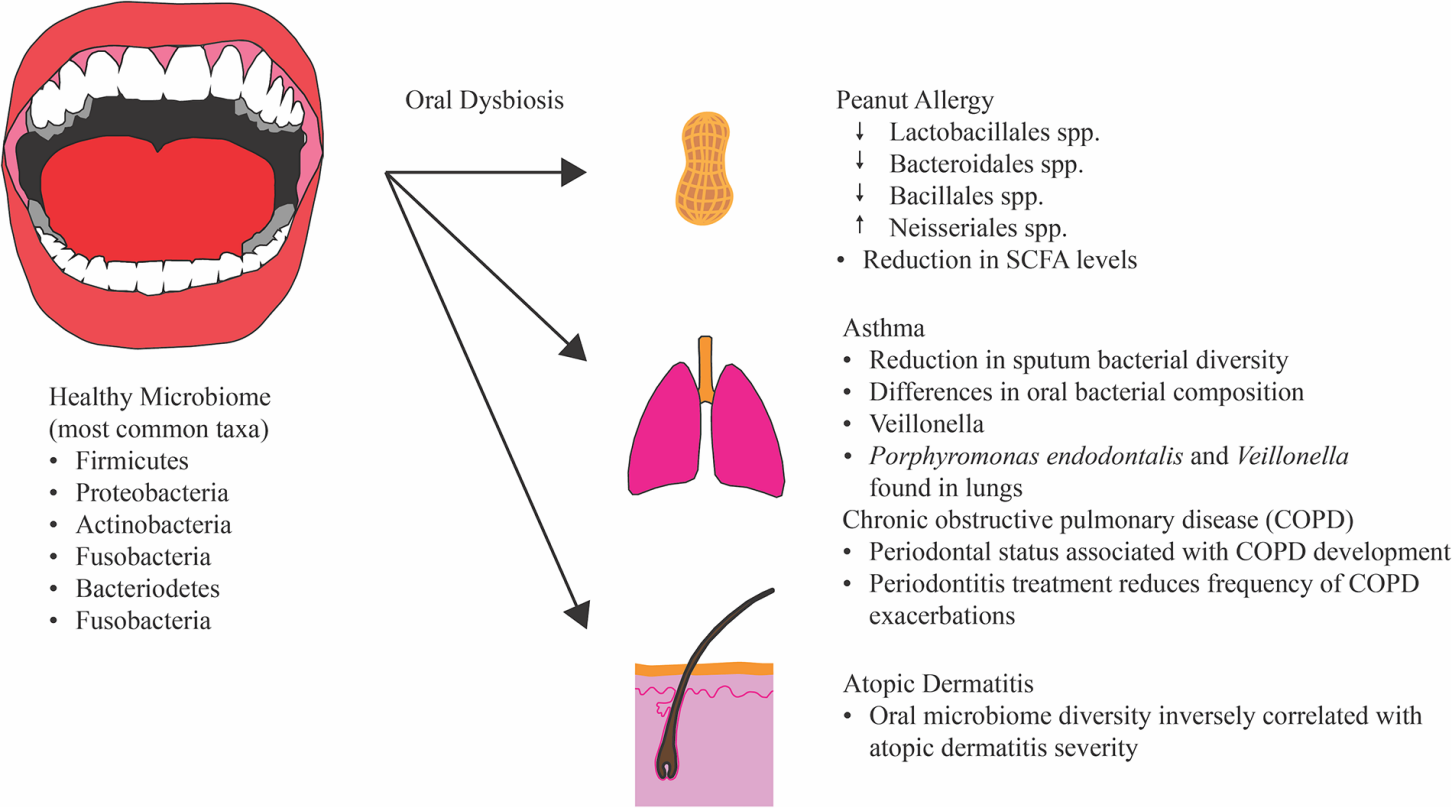
Alterations in oral microbiota composition is associated with allergic and respiratory diseases. Disruptions in oral microbiota composition have been associated with peanut allergy, asthma, chronic obstructive pulmonary disease, and atopic dermatitis, suggesting oral microbiota have an immunomodulatory role with allergy and respiratory development.

Dzidic et al. found associations between oral bacteria composition and allergy and asthma development during a 7-year longitudinal study in which DNA from salivary samples was isolated from children at 3, 6, 12, 24 months, and 7 years of age who were developing allergic symptoms and sensitizations (*n* = 47) and from children with no clear allergic symptoms (healthy) up to 7 years of age (*n* = 33) (Figure 3) (32). Sensitization was determined by at least 1 positive skin prick test and/or detectable circulating allergen-specific IgE antibodies. Skin prick tests used in this study were performed at 6, 12, 24 months, and 7 years of age on the forearm of the children with potential allergens including: egg white, fresh skimmed cow milk, and standardized cat, birch, and timothy extracts. The allergic diseases considered in their study were eczema, gastrointestinal allergy, allergic rhinoconjuctivitis, and allergic urticaria. Levels of circulating IgE antibodies specific to egg white, cow's milk cod, wheat, peanut, and soybean were also analyzed at 6, 12, and 24 months of age. Asthma diagnosis was determined by either a doctor diagnosis and asthma symptoms and/or medication within the last 12 months, or the presence of wheezing or nocturnal coughing and a positive reversibility test with spirometry. Using next-generation sequencing (NGS), the authors determined that salivary bacterial diversity was significantly lower in 7-year-old children with allergies compared to healthy 7-year-olds. The same is true for 7-year-olds with asthma compared to healthy children. Specifically, they amplified the 16S rRNA gene using universal degenerate primers prior to sequencing and used bioinformatic and statistical software packages to assess bacterial alpha-diversity (diversity within the same sample). The authors identified several genera- and species-specific associations in children with allergies and asthma at different stages of development. Increased relative abundance of the genera *Bacteroides* at ages 3 months and 7 years were associated with the development of allergic diseases, as were increased levels of *Streptococcus lactarius* and *Gemella haemolysans* at 7 years of age. Increased levels of *Alloprevotella* at 1 year of age and *Staphylococcus* at 2 years of age were associated with asthma development. They also identified several *Streptococci*, including *S. sanguinis* and *S. mitis*, that were found in greater abundance in asthmatic children. Their work supports the hypothesis that the colonization of a diverse microbiota during early childhood is necessary for inducing and maintaining tolerance (32, 96). However, Dzidic et al. state that their results suggest that the abundance of oral microbiota, such as *G. haemolysans*, *Lactobacillus gasseri*, and *L. crispatus*, was more important than diversity in allergy development.

Another study by Durack et al. identified associations between sputum and oral microbiota composition with immunological features in atopic asthmatic (AA) and atopic non-asthmatic (ANA) subjects (97). 32 AA, 18 ANA, and 16 non-atopic healthy control subjects were included in their study, and the bacterial diversity and abundance of induced sputum and oral wash samples from the subjects were assessed using NGS of the V4 region of the 16S rRNA gene. Type 2 (T2) airway inflammation was also evaluated by measuring expression levels of 3 bronchial epithelial genes (*CLCA1*, *SERPINB2*, and *POSTN*) induced by IL-13 and calculating a “3 gene mean” score (TGM) score. 22 of the AA subjects were classified as T2-low (TGM < 1.117) in this study. The authors found that sputum burden was inversely associated with the bronchial expression of the T2-related genes and that a cluster of T2-low asthmatic subjects had elevated proinflammatory lung cytokine levels and lower sputum bacterial diversity (Figure 3). Differences in relative abundance of bacteria genera, such as *Streptococcus*, *Moraxella*, *Neisseria*, and *Lactobacillus*, were associated with asthma and others with atopic status (*Granulicatella* and *Aggregatibacter* among others). The authors also noted that differences in oral taxa between the groups seemed more reflective of atopic status (97). Additionally, after asthmatic subjects were treated with inhaled corticosteroids (ICS) for 6 weeks the compositional structure of sputum microbiota demonstrated significantly greater deviation from baseline in ICS non-responsive subjects than in ICS responsive subjects.

Chronic obstructive pulmonary disease (COPD) and atopic dermatitis are two inflammatory diseases that are not considered allergic diseases but share immunological pathways and risk factors to allergic diseases and are worth mentioning briefly. COPD is another chronic inflammatory disease that typically develops and progresses in response to noxious particles and gases, and its development and exacerbation have been associated with severe periodontitis in recent publications (Figure 3) (98–100). Associations between periodontal status and the risk of COPD development remained significant in these studies even after adjustments for other possible risk factors, such as years of smoking, smoking intensity, body mass index, and age (99, 100). Treatment of periodontitis was also associated with reduced frequencies of COPD exacerbations, according to a systematic review performed by Kelly et al. (98). However, a causal link between periodontal status and COPD development has yet to be established. Atopic dermatitis (AD), commonly referred to as eczema, is a skin disorder which results in itchy and inflamed skin lesions. The pathogenesis of AD includes type 1 IgE dysregulation, disruption in skin barrier function which may lead to infections, and cell-mediated immune dysregulation (101). The microbial diversity, assessed by NGS, within the mouth of AD patients (AD, *n* = 83) was found to be inversely correlated with AD severity in a recent study by Li et al. (Figure 3) (102). Additionally, they observed that 53 bacterial genera were significantly different in the oral cavity of AD patients (*n* = 83) compared to healthy patients (*n* = 77). However, a mechanism by which the oral microbiota composition may influence atopic dermatitis severity is still unclear. A possible explanation for the association between oral microbiota composition and AD severity is that systemic inflammation due to AD itself influences oral mucosal immunity and, consequently, oral microbial colonization rather than the reverse direction. Nevertheless, more evidence is needed to establish any sort of causality and directionality.

A study published in 2021 by Ho et al. investigated oral microbial, metabolic, and immunological associations with peanut allergy (34). The study included 56 patients with peanut allergy and 45 non-allergic subjects, and 16S rRNA gene sequencing was performed on salivary samples to assess oral microbiota composition. SCFA metabolite and oral secreted cytokine levels were measured using liquid chromatography/mass spectrometry and multiplex assays, respectively. The authors found that the oral microbiota of individuals with peanut allergy were both lower in phylogenetic diversity and different in composition. Specifically, the oral microbiota of peanut-allergic subjects was characterized by reduced levels of Lactobacillales, Bacteroidales, Streptophyta, and Bacillales. Increased levels of Neisseriales spp. were also observed. Thus, a distinct oral microbiota composition was seen in subjects with peanut allergy (Figure 3). The oral microbiota composition of peanut-allergic subjects (*n* = 49) was also associated with a statistically significant reduction in oral SCFA levels in comparison with non-allergic subjects (*n* = 39), including: acetate, butyrate, and propionate. The authors also observed an elevation in IL-4 secretion in peanut-allergic subjects. SCFAs are bacterial fermentation products that modulate immune responses (103). The authors also noted that decreased abundances of *Prevotella* spp. and *Veillonella* spp. in peanut-allergic subjects correlated significantly with reduced oral SCFA levels. Increased abundance of *Neisseria* spp. observed in peanut-allergic subjects was positively correlated with a Th2-skewed oral immune milieu (34).

Evidence suggests that *Lactobacillus* spp., such as *L. crispatus* and *L. gasseri* that can colonize oral mucosal epithelial, serve as immunomodulators of adaptive and innate immune responses and alleviate symptoms in individuals with allergic rhinitis (104, 105). It is known that microaspiration of saliva or nasopharynx secretions contribute to the translocation of oral microbiota to the respiratory tract. However, microaspiration frequency increases during lung disease, and a recent study found that increased presence of oral bacteria, such as *Prevotella* and *Veillonella*, can lead to increased Th17 inflammatory responses in the lungs (106). In fact, Espuela-Ortiz et al. evaluated the oral microbiota composition, using 16S rRNA gene sequencing, of African American children and young adults with asthma (*n* = 57) and non-allergic, non-asthmatic control subjects (*n* = 57) and observed that *Veillonella* was predominant in asthmatic subjects (33). Moreover, a study done to analyze the lung microbiota of asthma patients treated with inhaled corticosteroids also observed the presence of oral bacteria, *Veillonella* and *Porphyromonas endodontalis*, in the lungs (Figure 3) (107). It is known that asthma medications such as inhalers impact oral microbiota composition, and this complicates the directionality of asthma-oral microbiota conclusions. Still, the observations mentioned in this section suggest that oral microbial composition and ectopic colonization may influence immune response sensitization and contribute to the development of allergies and respiratory disease.

A goal of several of the studies mentioned in this section was to identify biomarkers of allergic disease development, and some genera of bacteria implicated in allergic disease development have been identified in multiple studies, including *Prevotella*, *Neisesria*, and *Streptococcus* (Table 1). The accumulation of additional correlation studies will aid in the identification of species-specific bacteria to study further for their role in allergy development. Associations are currently limited, and more work is needed to establish causal relationships. Additionally, the ambiguity of correlation studies between oral microbiota composition and allergy development is a challenge for this field of research as the directionality is sometimes not clear and some subjects were receiving ICS treatment or probiotic supplementation either during or 6 months before the beginning of the study (32, 34, 97, 107). Also, not all the mentioned studies agree in their findings. For instance, Ho et al., in their peanut allergy study, obtained oral salivary samples from 26 non-food allergic subjects with physician-diagnosed asthma, allergic rhinitis, and/or atopic dermatitis and found no significant differences in alpha-diversity or beta-diversity (variation in bacterial community between samples) between healthy controls and atopic controls without food allergy. Also, significant differences in SCFA levels observed in peanut-allergic subjects were no longer present in the atopic controls without food allergy. Though the size of this cohort is slightly smaller than those in the Dzidic, Durack, and Li studies, these discrepancies highlight the inconsistency of oral microbial associations with allergic diseases and other inflammatory diseases.

## Potential therapeutics to treat oral dysbiosis

**Figure 4:**
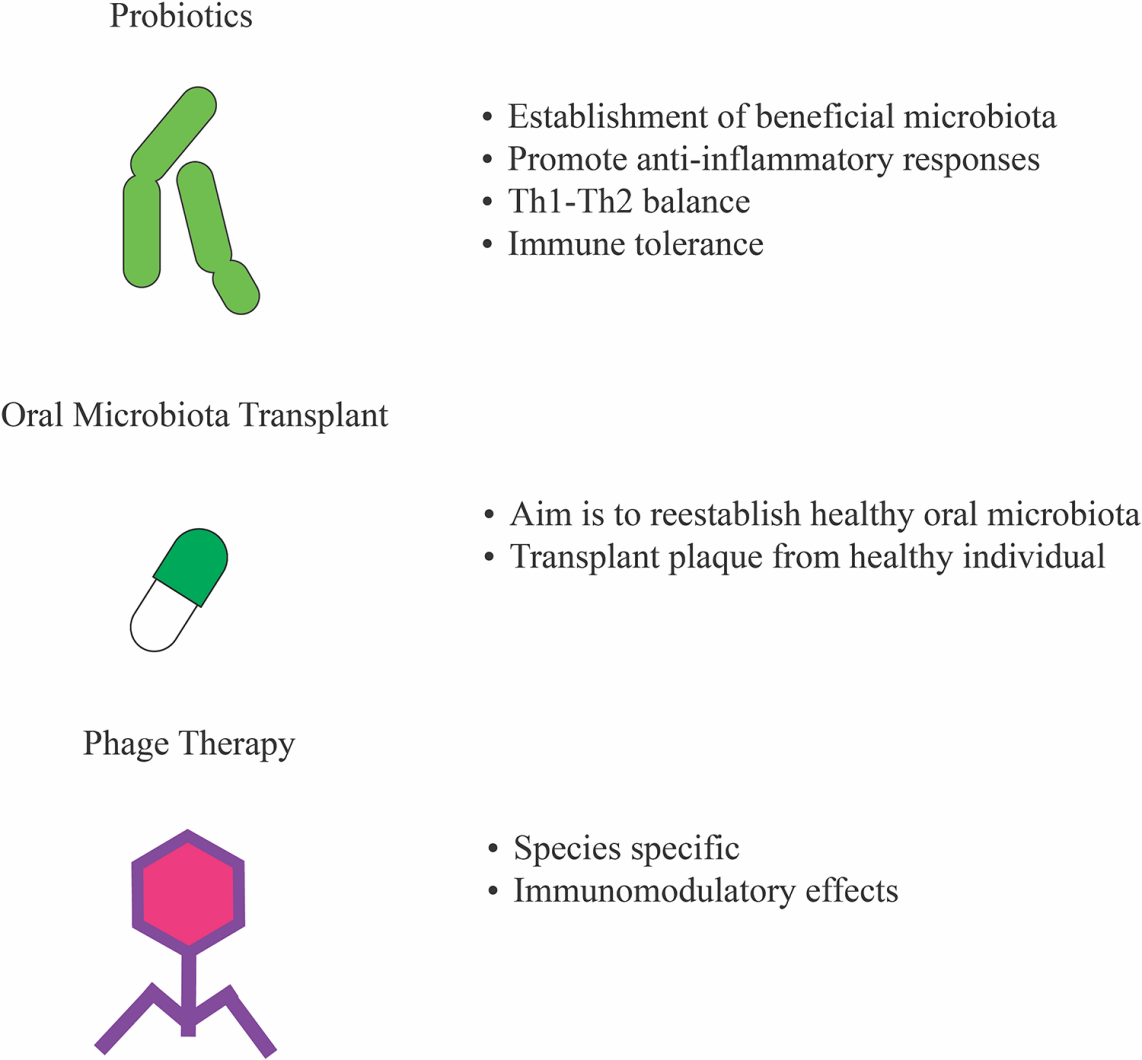
Potential therapeutics to treat oral dysbiosis. Suggested treatments to manage oral microbial dysbiosis to ameliorate diseases and allergies since alterations in microbiota composition is associated with immune sensitization and inflammatory disease.

The implication of oral microbiota influencing the development of allergies and other inflammatory diseases brings to question what can be done to establish healthy oral microbiota to ameliorate disease development. Currently, guidelines to treat oral diseases such as gingivitis and periodontitis, which may influence inflammatory and allergic diseases, rely on mechanical disruption of dental plaque which is generally efficacious in preventing disease progression. The administration of antibiotics or other antimicrobials is also recommended to treat bacterial infection during periodontitis (108). Recently, researchers have investigated other therapeutics to treat oral dysbiosis and infections including the use of probiotics in randomized controlled clinical trials as reviewed by Schlagenhauf and Jockel-Schneider (108). Here, we briefly mention some of those potential approaches (Figure 4).

Probiotics are live microorganisms known to be beneficial as they contribute to the re-establishment of healthy microbiota (109, 110). Probiotics are also associated with boosting immunity and metabolism (111). In the case of immunity, some probiotic strains can promote anti-inflammatory responses such as *Lactobacillus acidophilus* and *Lactobacillus casei* which promote Th1-Th2 balance, *Lactobacillus rhamnosus* which modulates wound healing, and *Akkermansia muciniphila* (112, 113). Probiotics also promote immune tolerance which may contribute to the prevention of allergies (114). Recent studies have demonstrated that probiotic bacteria, such as lactobacilli, can colonize the oral cavity and contribute to healthy microbiota reestablishment when used in products in contact with the mouth and not when ingested (115–117). However, results from randomized controlled clinical trials up to 2021 (36 total) evaluating the efficacy of probiotics reducing gingival inflammation in gingivitis and periodontitis patients were not consistent (108). These clinical trials varied in their dosage and application of the probiotic with some using the probiotic treatment as an adjunct to mechanical plaque removal and others using it as the only intervention. However, even studies with similar methods had contradicting results. Some studies report enhanced pocket closure in periodontitis patients receiving probiotic treatment as an adjunct to mechanical plaque removal when compared to the placebo group, while other groups failed to detect any significant additional benefit. The same was true for gingivitis patients given the probiotic as an adjunct. Nevertheless, the efficacy of probiotic use to treat oral dysbiosis can still be argued for in cases where the concept of strict mechanical plaque control fails, as supported by the positive results seen in trials where probiotic treatment was the only therapeutic measure given to individuals with chronic gingivitis or periodontitis and habitual poor oral hygiene (108).

Oral microbiota transplants (OMTs) have been suggested as a means to treat oral microbial dysbiosis (118). Inspired by the fecal microbiota transplants (FMT), OMTs involve the transfer of plaque from a healthy individual to a patient with gum disease (119, 120). FMTs have shown promising results in treating allergies such as peanut allergy by restoring healthy microbiota, and future studies can focus on the possibility to treat not only dysbiotic microbiota in the oral cavity but also aid in the prevention of allergies. However, no published study has tested the feasibility and efficacy of this treatment.

One of the more recently considered treatments to treat oral dysbiosis is bacteriophage-based therapy. Bacteriophages are viruses that target bacteria, and recent studies have suggested their potential role to reestablish beneficial microbiota and inducing immunomodulation (121). Bacteriophages have the unique ability to target specific bacteria, a characteristic that can be genetically modified (122). However, phage-resistant strains of bacteria may sometimes persist, and the targeting of one bacterial species may induce quantitative shifts in the population of another as microbiota communities are interactive (123). Bacteriophage therapy studies have shown anti-inflammatory effects by reducing levels of C-reactive protein and promoting the up-regulation of the anti-inflammatory cytokine IL-10, and the downregulation of TLR4 (124, 125). The use of bacteriophage-based therapy to alter oral microbiota composition remains an active area of research with many questions still needing to be addressed.

## Conclusions and future directions

It's clear that human microbiota influence immune system development and training, and correlations between oral microbiota composition and sensitization contributing to inflammatory diseases and allergic conditions have been made recently. Indeed, alterations in oral microbiota composition has many implications in chronic inflammation and other diseases. Oral microbiota composition has been associated with systemic inflammation and diseases ranging from cardiovascular disease to colitis and neurodegenerative disorders. Regarding allergic diseases, determining associations and mechanistic links between oral microbiota and allergic disease development is a novel field that is providing insight into risk factors for allergy development and possible therapeutic targets. Impaired barrier function, altered organ function, and susceptibility to infection caused by chronic allergic reactions and other inflammatory conditions, including chronic rhinitis and asthma, highlight the importance of this research. More research is needed to establish causal relationships and mechanisms between oral microbiota composition and the development of inflammatory diseases and allergic conditions. As we better understand how microbial composition in the oral cavity plays an immunomodulatory role, it may be possible to develop improved and novel therapeutics to treat or prevent the development of these inflammatory diseases and allergic conditions.
